# A three‐pronged approach that leans on Indigenous knowledge for northern fish monitoring and conservation

**DOI:** 10.1111/eva.13146

**Published:** 2020-10-31

**Authors:** Ella Bowles, Kia Marin, Pamela MacLeod, Dylan J. Fraser

**Affiliations:** ^1^ Concordia University Montreal QC Canada; ^2^ Department of Biology Irving K. Barber Faculty of Science University of British Columbia Okanagan Kelowna BC Canada; ^3^ Cree Nation of Mistissini Mistissini QC Canada; ^4^ Present address: 1177 Research Road Kelowna BC V1V 1V7 Canada

**Keywords:** bioinformatic filtering, body size, genomics, harvesting, Indigenous knowledge, size at age, walleye

## Abstract

Investigating whether changes within fish populations may result from harvesting requires a comprehensive approach, especially in more data‐sparse northern regions. Our study took a three‐pronged approach to investigate walleye population change by combining Indigenous knowledge (IK), phenotypic traits, and genomics. We thank Larson et al. (2020) for their critiques of our study; certainly, there are aspects of their critique that are warranted and merit further investigation. However, we argue that their critique is over‐stated and misleading, primarily given that (a) one of three prongs of our research, IK, was dismissed in their assessment of our study's conclusions; (b) our Bayesian size‐at‐age modeling should help to mitigate sample size issues; (c) their re‐analysis of our size‐at‐age data does not actually refute our results; (d) genomic changes that we observed are nascent; (e) the data file that Larson et al. (2020) used for their genomic re‐analysis was not correct; and (f) criteria that Larson et al. (2020) use for their genomic re‐analysis were not properly justified.

We thank Larson et al. (2020) for their critiques of our study, *Size reductions and genomic changes within two generations in wild walleye populations: associated with harvest*? (Bowles et al., 2020). The authors rightly point out that studying the genetic and evolutionary effects of harvesting on wild populations is a complicated, intricate process. We agree that alternative explanations highlighted by Larson et al. (2020) may influence the interpretation of trends observed in our study (e.g., recruitment, density, environmental conditions, the importance of careful bioinformatic filtering, the need for large sample sizes for phenotypic analyses). This is why (a) we included many of these alternatives in our paper's discussion and (b) we were careful to make the paper's conclusions suggestive, not definitive. Furthermore, cognizant of the fact that our study was conducted over a relatively short period of time in which evolutionary/genetic changes could take place (~1–2.5 generations for the study species and location), we repeatedly emphasized throughout the paper that the genomic changes observed were nascent. As a result, we take issue with the commentary authors' inference throughout their critique that we claim a causal relationship between harvest and the phenotypic/genomic changes observed.

We note that interpretations of our research findings included results from surveys of harvest trends based on local Indigenous knowledge (IK). Larson et al. (2020) barely acknowledge IK in their critique of how our study was conducted. In addition, Larson et al. (2020) reached a conclusion with their independent reassessment of our phenotypic analysis that does not refute our tentative conclusion. Moreover, several aspects of the bioinformatic filtering used for their critique are either incorrect, do not replicate our analyses, or have no basis.

Prior to addressing the critique of our analyses, we note that Larson et al. (2020) erroneously claim that we show that harvest of walleye in the study system (Mistassini Lake, QC, Canada) was 54% higher in 2003, a time that corresponds to when our historical samples were obtained, but varied little in other years, 1997, 2011 and 2015 (Table S2 (Bowles et al., 2020)), despite increases in the local Indigenous population. These values reflect only the reported harvest from non‐Indigenous fishers. To be clear, they do not account for the unreported harvest of Indigenous fishers who comprise the majority of fishers in the lake, nor secondary mortalities from fishing derbies that are popular locally. Therefore, this information cannot be referenced as total harvest in this system.

## 1 INDIGENOUS KNOWLEDGE: NOT CONSIDERED IN THE CRITIQUE

In our study, we used a three‐pronged approach for assessing walleye population change by combining IK, phenotypic traits, and genomics. Indigenous knowledge was an important component of our work and factored in greatly in the overall interpretation of what the collective results could signify. Our research was conducted in a remote area of northern Canada where (a) data are usually sparse and not easily collected; and (b) conservation recommendations have to be made despite data uncertainties. Indigenous knowledge can provide much more holistic assessments than Western science (IPBES, 2019; Levac et al., 2018; McGregor, 2004), over greater timescales (Gagnon & Berteaux, 2009), and arguably provides much better information surrounding adaptation (Fraser et al., 2006; Marin et al., 2017; Polfus et al., 2016; Smith & Sharp, 2012). The important collective knowledge base that included IK was not considered in the critique of Larson et al. (2020) and was dismissed by their statement that we have no quantitative evidence of harvest or exploitation rates. The IK provided evidence that fishing was linked to reduced body sizes and reduced catch rates, and highlighted concerns about how much harvesting was taking place in spawning rivers near the community in southern Mistassini Lake (Table 3 of Bowles et al., 2020). The available scientific data corroborated these Indigenous concerns about reduced body sizes and nascent life history and genomic changes in populations near the community where most fishing takes place.

## 2 BODY SIZE AND SIZE‐AT‐AGE ANALYSES

Regarding our body size analysis, Larson et al. (2020) argue that we did not include factors other than river, year, and sex in our models; for example, we did not include environmental variables, quantifiable information on harvest or exploitation rates of the populations in question, or size selectivity of harvest. However, we did include quantitative information on size selectivity of harvest in the discussion, with larger fish being removed by fishers. We discuss the evidence regarding the harvest/exploitation results issue in the IK section above and the last paragraph of our conclusions because it relies on a synthesis of the evidence that we collected. Otherwise, data were simply not available for other environmental factors (though this is being addressed in the system now), we discuss many of these aspects in our conclusion, and we discuss making conclusions/recommendations based on sparse data in the IK section above. Lastly, we emphasize that our models showed consistent results of reduced body size for 3 years in the south, relative to an earlier time‐point, while we did not see the same signal in the reference, less‐harvested northern location. Our sample sizes seem large enough (dozens to hundreds of samples) to estimate whether body sizes are different between two categories, just as we would use in a basic ANOVA.

Regarding our size‐at‐age analysis, Larson et al. (2020) argue that the data are insufficient to examine patterns and secondly that they re‐analyzed our data and found that size‐at‐age declined across both the more exploited southern populations and the northern less‐harvested reference population. Regarding their first point, while our sample sizes are clearly not ideal, we estimated observation error explicitly in our Bayesian model, and it was hierarchical so uncertainty was propagated between the groups. Low sample sizes would generally increase the observation error and estimation uncertainty, but information partially pooled from the other groups can help to anchor those estimates. Thus, the Bayesian hierarchical model should help to trade‐off estimation of effects against their sample sizes within the groups. Helser and Lai (2004) remark about sample sizes that, for a three‐parameter von Bertalanffy growth model, one needs at least five data points (age classes in their case) to justify including a population in the model hierarchy, three to estimate the growth curve, leaving two degrees of freedom. Wilson et al. (2018) also conducted a simulation with unbalanced sample sizes on a similar growth model, and they could recover relatively unbiased estimates of growth parameters even with unbalanced data and the occasional low sample sizes. Maas and Hox (2005) evaluate sample sizes in multilevel models, finding that having at least 5 samples per group (termed ‘group size’) is hardly any worse than having >25 samples. That being said, Maas and Hox (2005) also recommend having at least 30 groups (which would be walleye populations in our case), so application to our findings must be taken with caution. Perch River in 2015 is our lowest sample size, with 6 size‐at‐age samples (five different ages). It is the most worrisome but hovers around the limit needed for analysis.

To clarify Larson et al.'s interpretation of our findings, they state that our finding that fish were 29.4 mm larger contemporaneously than historically refutes the hypothesis that harvest negatively influenced growth over time. We note that fish being 29 mm larger was the case if all other factors were held equal and thus cannot be associated with the result that tested our hypothesis (the history term). Additionally, we discuss the point that Larson et al. (2020) make regarding the proportion of the posterior mass that falls below zero in the last paragraph of our conclusion.

Regarding the commentary authors' second point, that size at age has decreased in both the south and the northern (reference) population, this could be possible, but does not invalidate our conclusions about the south. Larson et al. (2020) state that our reference population is “unfished.” We do not claim this. In fact, fishers and community members are beginning to raise concerns regarding that population now as well, and these began sometime between 2015 and the present. We refer to Takwa River as having been “largely unaffected by fishing” for the time period of our study. Our body size models indicated that body size had been consistent between time‐points in the northern (Takwa) river, and more nuanced models may find something slightly different. It could indeed be the case that size at age is decreasing in Takwa River as well; in fact, Larson et al.’s finding that fish in the south decreased by more than those in the north makes sense.

## 3 GENOMIC ANALYSES

Larson et al., (2020) posit that our three main genomic conclusions are due to incorrect bioinformatic processing and inadequate data filtering, namely (a) genetic homogenization between two southern rivers, (b) genetic structure between time‐points in the Chalifour population, and (c) putative signatures of selection between time‐points in the southern rivers. We acknowledge of course that there are many ways to filter bioinformatic data and that filtering can have a considerable impact on conclusions reached. However, before discussing Larson et al.'s, (2020) conclusions, it is important to note that they used a dataset without outlier loci removed for their population structure analyses, while we removed outlier loci. In addition, for our within‐river between‐year pairwise outlier analyses we ran separate *stacks populations* (Catchen et al., 2011, 2013) runs for each contrast, requiring that all loci be present in both populations (historic and contemporary time‐points) in order to use them. It is erroneous for Larson et al. (2020) to re‐assess our within‐river, between‐year contrasts using the larger file containing all populations (and for which loci had to be present in only six or eight populations) because many loci we used will have been different.

Addressing the first technical critique, we found possible homogenization between Perch River and Icon River over a 1–2.5 generation time span. However, this was a shallow result, and we discussed alternative explanations. Regardless, Larson et al. (2020) discuss that all individuals in Perch 2003 had >50% missing data (their Fig S1). They suggested that this resulted from the combination of the ‐r and ‐p filers that we used in the *populations* module of *stacks*: ‐r 0.8, which requires that 80% of individuals within a population must contain a locus to use it; and then ‐*p* = 6/8 or 4/6 populations, whereby loci must be present in six out of eight populations (or 4/6 when Icon‐Perch were merged) in order to use them. These filters are reasonable, but the amount of missing data in Perch is concerning. The alternative to not including Perch in our analysis, however, was that we could not make inference about this population at all. Historic Perch River tissue samples were degraded, and this likely affected how well they sequenced. Additionally, we emphasize here that our paper discusses the possibility that a change in population structure did not occur.

The other major concern raised in the commentary is that there are some loci in the dataset with very negative F_IS_, and these generally correlate with very high F_ST_; this pattern shown in Figure 5 of Larson et al. (2020) is indeed strange. We set our F_IS_ filter at <−0.3. The *stacks populations* sumstats file reflects that somehow at least some of the loci that should have been filtered using our blacklist with loci having F_IS_ <−0.3 were not filtered correctly; 138 loci remained. There was an issue with blacklists leaking in at least one version of *stacks*, and we may have been using the pipeline in that iteration. However, as Larson et al. (2020) point out, only about half of the loci that they identified as having very negative F_IS_ deviated from what they term as typical patterns using HDplot, and then a large number of these loci fell into a normal allele ratio. They then replicated some of our analysis, but using different F_IS_ filtering criteria, filtering for missingness data and excluding loci with F_IS_ < −0.2 and then <0. It is strange that they did not replicate our −0.3 filter (which has been used elsewhere in comparable studies (Allen et al., 2017; Laporte et al., 2016; Morris et al., 2018)), and there is no justification for their cutoffs (as they say). F_IS_ will naturally fluctuate around zero in a randomly mating population, with positive F_IS_ values possibly indicating selection. Lastly, and regardless, Larson et al. (2020) claim that structure disappears with the −0.2 F_IS_ cutoff (their Fig 4). There is certainly less structure there, but we would claim that structure still exists (both evident in their Fig 4, and F_ST_ at *p* < .01 is not significant, but *p* < .05 was not presented). In sum, there is a concerning pattern with some loci that the commentary authors have found; however, it is not clear whether these problematic loci were removed in our analysis since we removed outliers and they did not, we used a unique pairwise file for within river contrasts, the rationale for their filtering is not clear, and even with their filtering population structure may still be present between years in Chalifour River.

## 4 CONCLUSIONS

Conclusively demonstrating that changes within fish populations are the result of harvesting can be difficult, as Larson et al., (2020) point out, and there are aspects of their critique that merit further investigation. However, we argue that their critique is over‐stated and misleading given that (a) one of three prongs of our research, IK, was dismissed; (b) there is sufficient evidence from the literature that our Bayesian size‐at‐age modeling should help to mitigate to some extent sample size issues; (c) the result from their re‐analysis of our size‐at‐age data does not actually refute our results; (d) genomic changes that we observed are nascent, (e) the data file that Larson et al. (2020) used for their genomic analysis was not correct; and (f) criteria that Larson et al. (2020) use for their genomic re‐analysis were not properly justified.

Beyond the technical critiques of Larson et al. (2020), they are clearly concerned that we lack sufficient evidence to falsify the hypothesis that harvest may be associated with the observed population changes, or to alternatively reject the null hypothesis that walleye populations can demonstrate considerable natural variation in growth from year to year (Bozek et al., 2011; Dembkowski et al., 2014; Hansen & Nate, 2014; Sass et al., 2004). In conservation biology, perhaps it behooves us to consider that large effects can happen and we do not want to falsely accept a null hypothesis (“power” or type II error) and worry slightly less about committing a type 1 error (rejection of a true null hypothesis) at an alpha of 0.05 (in frequentist statistics). We contend that this is especially important in our research context, because walleye are slow‐growing and maturing (Bozek et al., 2011; Venturelli et al., 2010) and known to be susceptible to overharvesting across the species range, especially in northern environments like Mistassini where growing seasons are shorter (Baccante & Colby, 1996; Colby & Baccante, 1996).

What would variation in size/growth from year‐to‐year mean for the average body length/mass of dozens/hundreds of samples to support three consecutive years of body sizes below a baseline reference period? It would mean either that growth was exceptionally poor for a number of consecutive cohorts or that larger fish are no longer in the dataset. Why would we expect the former to be the case for all populations during the same years? Along these lines, regarding our Bayesian models, Larson et al. (2020) state that the size difference that our models uncovered between time‐points was small (13.7 mm), with a confidence interval that overlapped zero with 13% of the posterior mass <0. The credible intervals used for Bayesian inference are fairly arbitrary (some use 80%, some 89% (McElreath, 2020), some use 95% to mirror the frequentist alpha of 0.05), but there are no discrete thresholds crossed for Bayesian inference as there is frequentist. Instead, Bayesian inference is a continuous process from 0% to 100%. In our case, there is substantial support (87% support) that the declining trend in size was different between locations.

Overall, if we were relying on one or two metrics, we would not feel confident in making any inference or potential association with harvest. However, we are not, we have evidence from IK, body size, size at age, and genomics that suggest that populations are changing and that harvest may be implicated in the changes observed.

## CONFLICT OF INTEREST

The authors declare no conflicts of interest.

## Data Availability

No data have been used for this article.
